# The effect of dietary supplementation with high- or low-dose omega-3 fatty acid on inflammatory pathology after traumatic brain injury in rats

**DOI:** 10.1515/tnsci-2021-0010

**Published:** 2021-02-04

**Authors:** Elise K. Black, Jack K. Phillips, Jack Seminetta, Julian Bailes, John M. Lee, John D. Finan

**Affiliations:** Department of Neurosurgery, NorthShore University HealthSystem, Evanston, IL, United States of America; Department of Pathology and Laboratory Medicine, NorthShore University HealthSystem, Evanston, IL, United States of America; Department of Mechanical and Industrial Engineering, University of Illinois at Chicago, Room 2035 Engineering Research Facility, 842 W Taylor Street, Chicago, IL 60607, United States of America

**Keywords:** omega-3 fatty acid, docosahexanoic acid, eicosapentaenoic acid, traumatic brain injury, dietary supplementation, neuroinflammation, neuroprotection

## Abstract

This study investigated dietary supplementation as a prophylactic for neuroinflammation following traumatic brain injury (TBI) in a preclinical model. Adult male Sprague-Dawley rats received 30 days of supplementation with either water or two dietary supplements. The first consisted of high-dose omega-3 fatty acid (O3FA) (supplement A) along with vitamin D3 and vitamin E. The second had the same ingredients at different doses with an addition of cannabidiol (supplement B). Rats were subjected to an impact TBI and then euthanized 7 days post-injury and neuroinflammation quantified by histological detection of glial fibrillary acidic protein (GFAP), a marker of astrocyte activation, and CD68, a marker of microglial activity. There was a trend toward increased GFAP staining in injured, unsupplemented animals as compared to sham, unsupplemented animals, consistent with increased activation of astrocytes in response to trauma which was reversed by supplement A but not by supplement B. The pattern of CD68 staining across groups was similar to that of GFAP staining. There was a trend toward an increase in the injured unsupplemented group, relative to sham which was reversed by supplement A but not by supplement B. CD68 staining in injured animals was concentrated in the perivascular domain. The consistency between trends across different measures of neuroinflammation showing benefits of high-dose O3FA supplementation following TBI suggests that the observed effects are real. These findings are preliminary, but they justify further study to determine the functional benefits associated with improvements in histological outcomes and understand associated dose-response curves.

## 1 Introduction

Traumatic brain injury (TBI) remains a pressing public health concern. Approximately 2.5 million people are diagnosed with TBI in U.S. emergency rooms every year [1]. Most of these injuries are classified as mild. However, recent findings suggest that even mild TBIs can have severe long-term consequences [2], particularly in the cases of individuals who sustain repeated mild TBI. Mild TBI diagnosis can increase the incidence of Alzheimer’s disease and Parkinson’s disease and can also cause a TBI-specific form of neurodegeneration called chronic traumatic encephalopathy in some patients [3,4,5]. In light of these concerning trends, there is an urgent need for interventions that can mitigate the morbidity associated with mild TBI, particularly among populations at elevated risk of mild TBI. These populations include military service members and athletes participating in contact sports.

TBI remains without an approved therapy. One reason for this is that the pathology of TBI proceeds rapidly after the impact [6]. Preclinical studies find that candidate therapies are most effective when applied immediately after or even before injury [7]. However, immediate treatment is clinically impractical because TBI occurs unexpectedly, and delays associated with diagnosis, transport, and management of other trauma sequelae are common.

The best strategy to avoid the detrimental short- or long-term effects of all TBIs, mild-to-severe, is through avoidance of the injury or primary prevention. TBI is too complicated for a narrow pharmaceutical approach. Approaches that target multiple aspects of TBI – secondary injury, repair, regeneration, and protection of the brain – are needed. For the latter approach, a nutraceutical is attractive. Effective interventions should also treat persistent symptoms associated with the long-term effects of TBI (post-concussive symptoms; e.g., memory disturbances, depression, headache) [8].

Studies have clearly shown that omega-3 polyunsaturated fatty acids (O3FA) such as eicosapentaenoic acid (EPA) and docosahexaenoic acid (DHA) are essential for proper brain development and function [9,10]. There have been numerous preclinical studies in rodent models that have shown the benefits of O3FA in reducing the pathology and negative outcomes associated with TBI, stroke, and spinal cord injury [11,12,13,14,15]. However, work on the underlying mechanisms is still unfolding [15,16]. Cannabidiol (CBD) has also shown the potential to mitigate TBI pathology in preclinical models [17,18,19]. Accordingly, this study tested two O3FA dietary treatments: one with high-dose DHA and one with low-dose DHA combined with cannabidiol (CBD) to examine their efficacy as prophylactic interventions against TBI. Vitamin D3 and vitamin E were also included at various doses in the hope that they would synergize with O3FA and CBD to optimize outcome. Vitamin D mitigates lipid peroxidation [20], which is an important pathology in neurotrauma [21]. Vitamin E protects cells from free radicals, which play an important role in neurotrauma pathology [22].

## 2 Method

### 2.1 Animals and dietary supplement information

About 48 male Sprague-Dawley rats were obtained from Charles River and received chow and water *ad libitum*. Their diet was supplemented with 270 µL of either water, supplement A or supplement B every day for 30 days before the injury. The 30-day treatment period was selected based on successful neuroprotection in similar prior studies from our group that used this treatment period and also on evidence in the literature that it takes at least 12 days for dietary changes to be reflected as changes in the fatty acid composition of the brain [11,12,13,24].

Table 1 details the composition of the supplements (product supplied by Trident Brands Inc.). The animals were evenly and randomly distributed across the following four experimental groups: sham injury without supplementation, an injury without supplementation, injury with supplementation A and injury with supplementation B.

**Table 1:** Supplement composition and dosing of animals (DHA = docosahexaenoic acid, EPA = eicosapentaenoic acid, CBD = cannabidiol)

Ingredient	Amount per mL	Amount per dose
**Supplement A**		
DHA	250 mg	67.5 mg
EPA	125 mg	33.8 mg
Vitamin D3	360 IU	97.2 IU
Vitamin E	9 IU	2.43 IU
**Supplement B**		
DHA	50 mg	13.5 mg
EPA	25 mg	6.75 mg
Hemp	14 mg	3.78 mg
CBD	2.5 mg	0.675 mg
Vitamin D3	100 IU	27 IU
Vitamin E	7.5 IU	2.03 IU


**Ethical approval:** The research related to animals’ use complied with all the relevant national regulations and institutional policies for the care and use of animals. All animal experiments were performed in accordance with The Guide for the Care and Use of Laboratory Animals, and all protocols were approved by the Institutional Animal Care and Use Committee at NorthShore University Health System [20] (Protocol Number: EH18-191).

### 2.2 The Marmarou impact acceleration injury model

The Marmarou model is well established to produce brain injuries as previously described [11,21]. Briefly, anesthesia was induced with inhaled 4% isoflurane and maintained with 2.5% isoflurane. The surgical site was shaved, and 1% lidocaine at 3 µg/kg was administered intradermally at the planned incision sites. A 3-cm midline incision in the scalp was made to expose the skull. A metal disk 10 mm in diameter and 3 mm thick was cemented to the skull using dental acrylic on the midline centered between bregma and lambda. Animals were then disconnected from the isoflurane and placed prone on a foam bed (Type E bed manufactured by Foam to Size, Inc., Ashland, Virginia) with the metal disk directly under a Plexiglas tube. A 450 g brass weight was dropped through the tube from a height of 2 m onto the disk attached to the skull. In some cases, apnea occurred immediately after impact. In these cases, animals were ventilated via a nosecone with 100% oxygen using a TOPO Dual Mode Ventilator (Kent Scientific Corporation) until spontaneous respiration was observed [22]. The animals were subsequently sutured under isofluorane anesthesia and returned to their housing. Buprenorphine was administered subcutaneously at 0.05 mg/kg every 8–12 h for the first 24 h after surgery for analgesia.

### 2.3 Histology

Inflammatory pathology was quantified by histological detection of glial fibrillary acidic protein (GFAP), a marker of astrocyte activation, and CD68, a marker of microglial activity. All animals were euthanized with a lethal dose injection of 120 mg/kg ketamine and 15 mg/kg xylazine 7 days after injury. The animals were immediately perfused transcardially with cold phosphate-buffered saline (PBS) to clear the blood, followed by 4% paraformaldehyde in Millonig buffer until fixation tremors ceased. The brain was dissected out of the skull and placed in 4% paraformaldehyde for 72 h, before being dehydrated in 30% sucrose until it lost buoyancy (3–5 days). It was then blocked, and 50-µm thick coronal cryosections of the forebrain was taken at locations 3.5–5 mm caudal to bregma.

For CD68 labeling, cryosections were subjected to temperature-controlled microwave antigen retrieval using a Pelco Biowave Microwave 34700 as previously described [23]. Sections were then incubated in primary mouse anti-CD68 monoclonal antibody (MA5-13324, Thermo Fisher Scientific) at a 1:150 dilution overnight in 0.25% Triton in tris-buffered saline (TBS). Staining was visualized using a Mouse Specific HRP/DAB Detection IHC Kit (ab64259, Abcam). For GFAP labeling, the tissue was incubated overnight with a primary unconjugated rabbit anti-GFAP polyclonal antibody (Z033429-2, Agilent Dako) at a dilution of 1:750. This was followed by a 2 h incubation in Donkey anti-Rabbit secondary antibody Alexa Fluor 555 at a dilution of 1:500(A-31572, Thermo Fisher Scientific). In both cases, the slides were mounted in Vectashield mounting medium (Vector Laboratories) containing DAPI that labeled the nuclei.

### 2.4 Microscopy and image analysis

**Figure 1:**
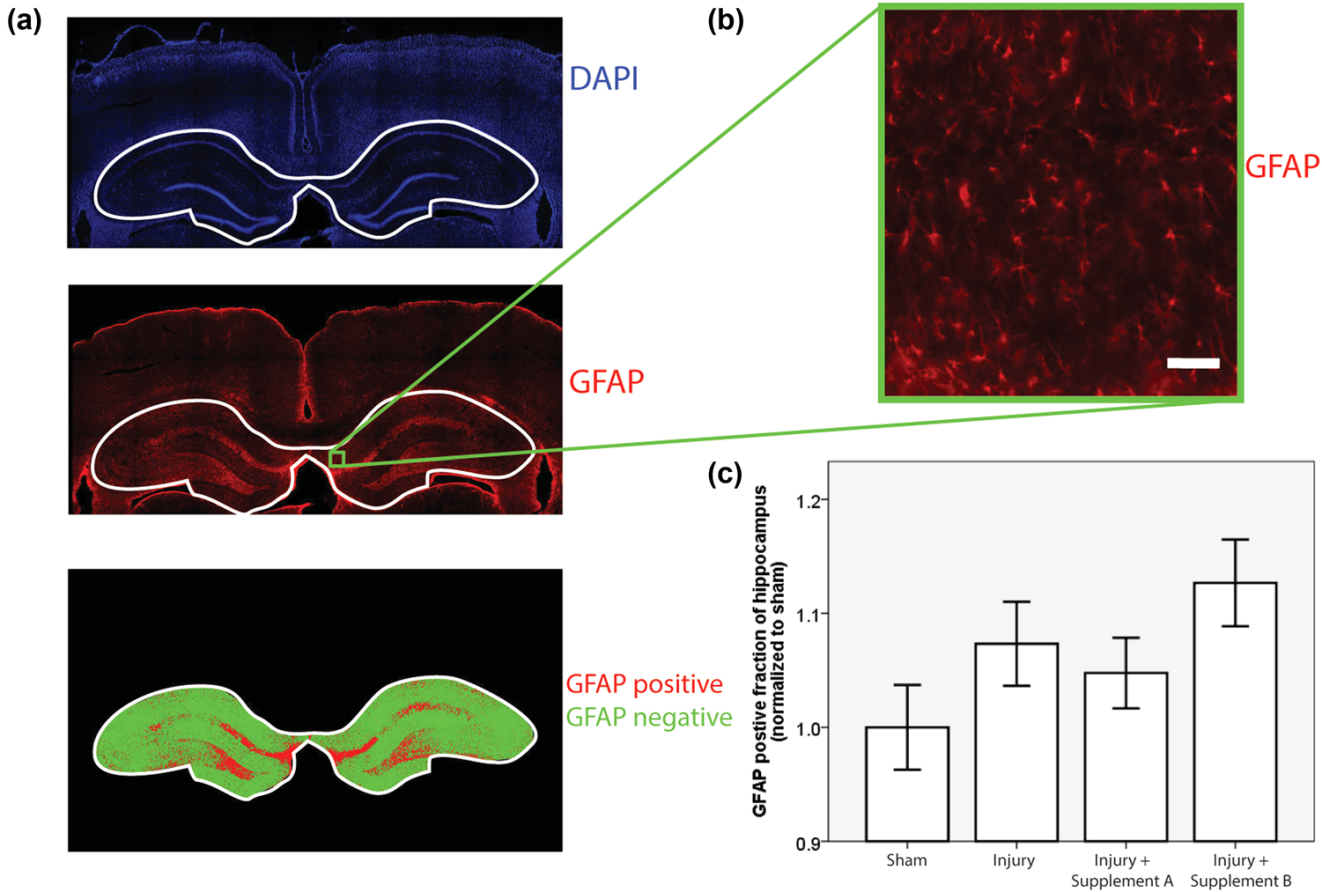
GFAP quantification. (a) The GFAP quantification workflow. A blinded operator outlined the hippocampus in the DAPI image. The hippocampal region in the GFAP images was then automatically thresholded to identify the GFAP-positive (red) and GFAP-negative (green) regions, (b) close-up view of GFAP fluorescence (scale bar = 50 µm), (c) mean values of GFAP positive fraction of the hippocampus by group, and normalized to sham values within each cohort (*n* ≥ 11, error bars = standard error, ANOVA *p* = 0.104).

For GFAP quantification, whole, fluorescently labeled, coronal sections of the forebrain were scanned with a 4× air objective in an automated fashion by a TissueGnostics Tissue Cytometer. One region of interest containing the hippocampus and adjacent cortex was then selected in each animal using the TissueFAXS software provided by the manufacturer. The hippocampus was the focus of histological investigation because of its critical role in learning and memory, functions that are often impaired after mild TBI. This region was scanned again with a 10× air objective with a numerical aperture of 0.5. GFAP fluorescence was captured with the rhodamine filter, and DAPI fluorescence was captured with the DAPI filter. A custom written, semi-automated Matlab (Mathworks Inc.) script was used to quantify the extent of GFAP fluorescence. This script presented the DAPI channel image to a blinded operator in a randomized sequence and allowed the operator to define the boundary of the hippocampus. This bounded region was then automatically thresholded in the GFAP channel using Matlab’s graythresh function, which employs the Otsu method [24]. The extent of the GFAP staining was then quantified as the ratio of the GFAP-positive area of the hippocampus to the total area of the hippocampus (Figure 1a). The process was performed by two different blinded operators, and their results were averaged. Staining was performed in four cohorts of animals distributed randomly across the experimental groups. The GFAP ratio in each animal was normalized to the mean GFAP ratio for sham animals in the same cohort to protect against the possibility of cohort-by-cohort variation in GFAP staining. The CD68 slides were microscopically inspected by an experienced, blinded clinical neuropathologist and scored on a zero to two scale based on the extent of the CD68 staining.

### 2.5 Statistics

The normality of the data was confirmed using a Shapiro–Wilks test. For normally distributed data, statistical significance was assessed using a one-way ANOVA test. Otherwise, statistical significance was evaluated using a Kruskal–Wallis test. Statistical tests were performed using SPSS software.

## 3 Results

Although animals weighed between 350 and 450 g at the start of the study, they continued to grow during the 30-day supplementation period and had an average weight of 529 g (SD = 46 g) at the time of injury (Table 2). There was no statistically significant correlation between the weight at the time of injury and the experimental group (ANOVA, *p* = 0.238).

**Table 2:** Average animal weights on the day of injury

Experimental group	*N*	Weight (g) ± SD
Sham	12	532 ± 48
Injury	12	548 ± 48
Injury with supplementation A	12	527 ± 55
Injury with supplementation B	12	510 ± 26
Total	48	529 ± 46

GFAP-positive cells were observed throughout the cortex and hippocampus and displayed the classic star-shaped morphology expected in astrocytes (Figure 1b). The Shapiro–Wilks test returned a *p* > 0.05 for all four experimental groups, indicating that the data were normally distributed. There was a trend toward increased GFAP staining in injured, un-supplemented animals as compared to sham, un-supplemented animals, consistent with increased activation of astrocytes in response to trauma. This trend was reversed by supplement A but not by supplement B. The *p*-value of the associated ANOVA test was 0.104 (Figure 1c).

**Figure 2:**
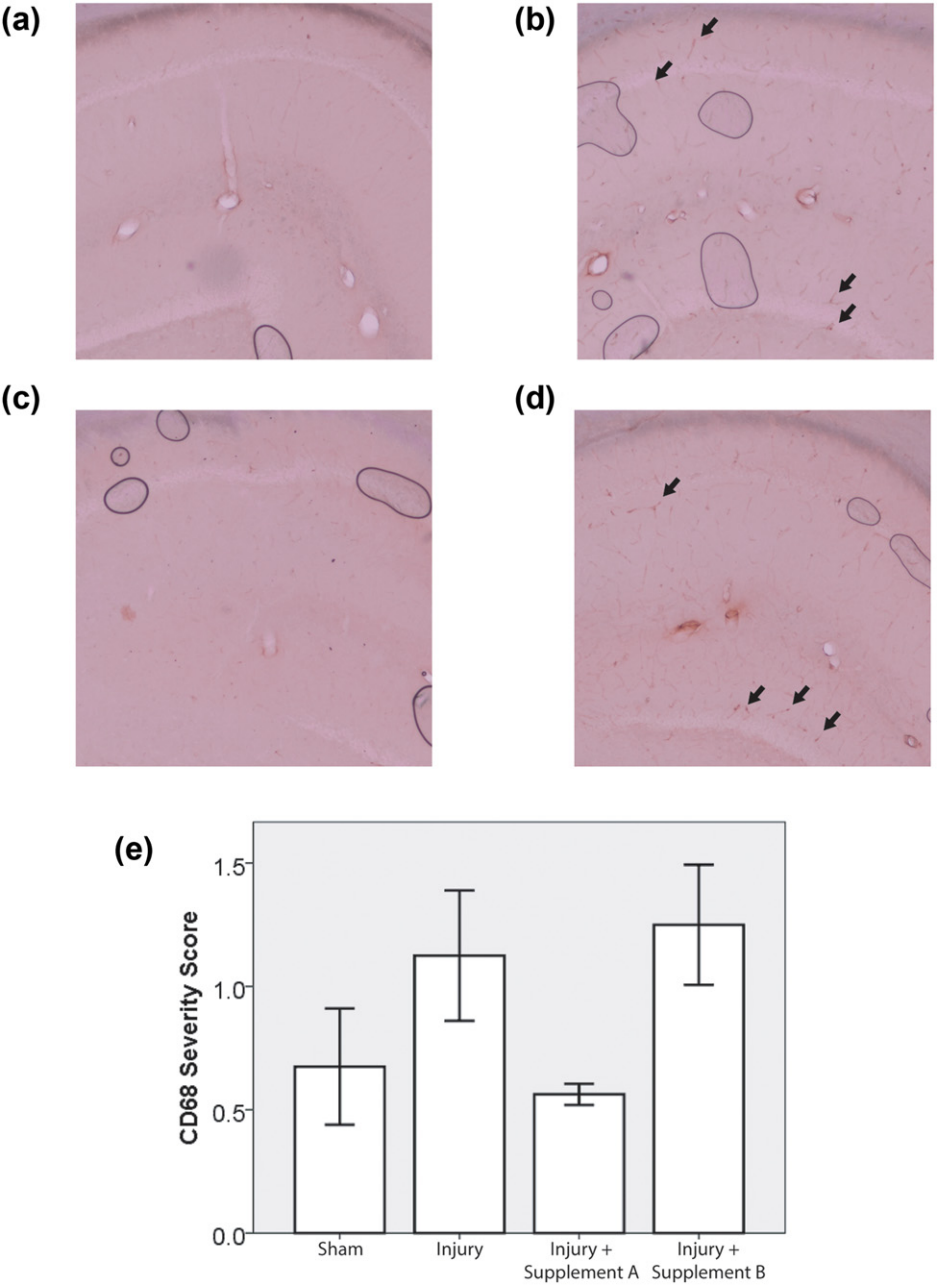
CD68 Semi-quantitative Scoring. CD68 staining in hippocampal sections from animals close to the average for the following groups: (a) Sham, (b) injury, (c) injury + supplement A, and (d) injury + supplement B. Arrows indicate positive staining in the perivascular domain, (e) summary statistics for the semi-quantitative scoring of CD68 pathology (*n* ≥ 5, error bars = standard error. Kruskal–Wallis test *p* = 0.057).

CD68 staining, where present, was concentrated in the perivascular domain (Figure 2b). The pattern of CD68 staining across groups was similar to that of GFAP staining. The Shapiro–Wilks test returned *p* > 0.05 for three of the four experimental groups but *p* was less than 0.05 for one group, the group treated with supplement A. Since one of the groups was not normally distributed, a Kruskal–Wallis test was used to determine the statistical significance of the trends. There was a trend toward an increase in the injured un-supplemented group, relative to sham. This trend was reversed by supplement A but not by supplement B. The *p*-value of the associated Kruskal–Wallis test was 0.057 (Figure 2c).

## 4 Discussion

This study observed trends suggesting an inflammatory response to mild TBI that was mitigated by pre-injury supplementation with supplement A but not with supplement B. The superior performance of supplement A compared to supplement B may arise from the fact that supplement A contains a four-fold higher concentration of DHA and EPA than supplement B (Table 1). Previous reports in the literature by our group and others have shown that DHA supplementation mitigates head injury pathology in preclinical rat models [11,12,13] in a dose-dependent manner [13]. However, it is worth noting that supplement A also contains more vitamin D3 than supplement B, and this may also influence therapeutic trends. Vitamin D improved outcomes in prior TBI studies in rat models and humans [25,26]. Vitamin D also synergized with progesterone treatment [26] and it may synergize with DHA treatment in this system. Further investigations will be required to address this possibility. In this study, CBD could not enhance the benefit of lower dose O3FA to match the therapeutic trend observed with the higher dose of O3FA or vitamin D3. These trends raise intriguing questions about the potential benefits of nutraceutical interventions for TBI and support further study of these agents in this model system to optimize dose and synergy effects.

The injury phenotype in this study was mild. The most likely explanation of the mild phenotype lies in the weight of the animals. Prior studies used rats weighing between 350 and 400 g at the time of injury, which corresponds roughly to the start of adulthood in rats. In this study, the animals began their period of dietary supplementation at a similar stage of maturity, consistent with the projected application of these supplements in young adults. Supplementation continued for 30 days, and the rats kept growing during this time, reaching an average weight of 529 g at the time of injury (Table 2). As rats grow larger, the skull becomes thicker and the head becomes more resilient to impact. This phenomenon is the most likely reason for the absence of axonal damage and apoptotic biomarkers. However, there was consistent evidence of neuroinflammation after trauma (Figures 1 and 2). Two biomarkers were used to measure neuroinflammation, GFAP and CD68. The pattern of variation in these biomarkers across experimental groups was strikingly consistent. In both cases, the levels rose with injury, and this trend was mitigated by treatment with supplement A but not with supplement B. This similarity occurred in spite of the fact that the two outcomes measured inflammatory pathology by staining different markers in different cells and quantifying that staining in different ways (semi-automated image analysis in the case of GFAP and visual inspection by a blinded neuropathologist in the case of CD68). ANOVA testing was used to test the statistical significance of the GFAP results, which were normally distributed, while a Kruskal–Wallis test was applied to the CD68 results, which were not normally distributed.

The *p*-value was 0.104 in the former case and 0.057 in the latter case. Neither score meets the conventional threshold for statistical significance (*p* < 0.05). In formal terms, the likelihood that the GFAP results reflect random variation is about 1 in 10 while the likelihood that the CD68 results are random events is a little more than 1 in 20. However, the likelihood of achieving this level of significance by chance simultaneously in two different outcomes indicating the same underlying pattern of pathology is much lower.

The mild injury phenotype observed is consistent with the goal of the study, which was to understand the capacity of nutraceutical approaches to mitigate the pathology of concussive and sub-concussive impact commonly encountered by athletes. Neuroinflammation lies at the heart of this process [27]. A drawback of this mild injury phenotype is that it hinders efforts to reach the commonly used statistical significance threshold of 0.05 and that threshold was not crossed for either of the neuroinflammatory outcomes reported. Nevertheless, the consistency between the trends observed across different measures of neuroinflammation suggests that the observed effects are real. These findings are preliminary but they justify further study to determine the functional benefits associated with improvements in histological outcomes and understand associated dose-response curves.
